# Semi-quantitative magnetic resonance imaging scoring of the knee detects previous injuries in professional soccer players

**DOI:** 10.1007/s00167-022-06897-5

**Published:** 2022-02-19

**Authors:** Goetz Hannes Welsch, Anna-Maria Behr, Karl-Heinz Frosch, Enver Tahir, Milena Pachowsky, Frank Oliver Henes, Gerhard Adam, Kai-Jonathan Maas, Malte Lennart Warncke

**Affiliations:** 1 Center for Sports Medicine, UKE Athleticum, University Medical Center, Hamburg-Eppendorf, Martinistrasse 52, 20246 Hamburg, Germany; 2 Department of Trauma and Orthopedic Surgery, University Medical Centre Hamburg-Eppendorf, Hamburg, Germany; 3 Department of Diagnostic and Interventional Radiology and Nuclear Medicine, University Medical Centre Hamburg-Eppendorf, Hamburg, Germany; 4 Department of Trauma and Orthopedic Surgery, University Hospital of Erlangen, Erlangen, Germany; 5 BG Hospital Hamburg, Hamburg, Germany

**Keywords:** Professional soccer, Knee MRI, Medical, Whole-Organ Magnetic Resonance Imaging Score

## Abstract

**Purpose:**

The medical examination (“medical”) is an important procedure in professional soccer since it has high economic relevance. In addition to clinical tests, magnetic resonance imaging (MRI) is used to assess joint health. In the present study, the reliability of semiquantitative knee MRI during the “medical” in professional soccer was tested, and its relationship with clinical data and days missed due to knee injury was observed.

**Methods:**

In this cross-sectional study, between 2012 and 2019, 69 newly assigned players (age 18–35 years) from a professional soccer club underwent MRI (3.0 T) of both knee joints during their “medical”. Reported knee injuries and previously missed days due to injury were obtained from player anamnesis and the “transfermarkt.com” database. Based on the established “Whole-Organ Magnetic Resonance Imaging Score” (WORMS), two independent radiologists graded the MRI results. Further evaluation was based on the mean score of both knees.

**Results:**

The mean WORMS for all subjects was 13.9 (median 10.5, range 0–61). Players with previous injuries had significantly higher scores than players without reported injuries (22.1 ± 17.7 vs. 8.9 ± 4.4, *p* < 0.002). Three outliers (previously undetected injuries) in the group of players without reported injuries were observed (6.7%). The WORMS was significantly correlated with a prior knee injury (*r*: 0.424, *p* < 0.0001) and days missed due to injury (*r*: 0.489, *p* < 0.001). Age was correlated with the WORMS (*r*: 0.386, *p* < 0.001). In a linear regression model, prior injury was the only significant predictor of a high WORMS (*p* = 0.001). The WORMS was a significant predictor of days missed due to injury (*p* < 0.0002) and prior injury (sensitivity: 78%, specificity: 91%, *p* = 0.006). The intraclass correlation coefficient was excellent (0.89).

**Conclusion:**

Semiquantitative knee MRI for WORMS determination during the soccer “medical” is a robust and reliable method. Prior injury, even in players without documented trauma, was detected by the WORMS, and previously missed days due to injury were correlated with the semiquantitative MR knee score.

**Level of evidence:**

Level III.

**Supplementary Information:**

The online version contains supplementary material available at 10.1007/s00167-022-06897-5.

## Introduction

Soccer, as the most popular sport in the world, with over 300 million active players and more than 200,000 professional players [6], is associated with repetitive joint injury as well as acute and chronic articular cartilage lesions [9, 10, 16, 19, 22]. Available meta-analyses have reported cartilage changes in a high percentage of active and former professional athletes, varying over a wide range from 2.4 to 75% [9, 18].

Articular cartilage lesions in the knee joint may produce typical symptoms, including pain, swelling, catching and locking, but may also be present in asymptomatic players [1, 23]. These degenerative changes can lead to reduced athletic performance as well as an early career end [3, 7, 11, 13]. Hence, severe injuries and secondary chronic changes, especially in the knee joint, can be key factors in the general assessment of soccer players.

The medical examination (“medical”) is an increasingly important procedure in sports medicine, with both high importance for maintaining the health of the players as well as high potential economic relevance due to the high transfer fees and increasing salaries that are being paid in professional soccer. Typically, no player complains during his medical about any musculoskeletal (or other) problems. Prior injuries or missed days that are not reported in the database “transfermarkt.com” are very often not reported to the examining physician. In addition to a clinical evaluation and athletic testing, diagnostic imaging, especially magnetic resonance imaging (MRI), is used to assess joint health. In morphological MRI of the knee, semiquantitative whole-organ scores can be used to objectively assess the status of the joint to the greatest extent possible [8, 15]. The Whole-Organ MRI Score (WORMS) of the knee is the most popular and most commonly used semiquantitative score [17, 20, 23, 26].

In the present study, the reliability of semiquantitative knee MRI during the “medical” in professional soccer was tested, and its relationship with clinical data and days missed due to knee injury was observed.

## Materials and methods

The study protocol was approved by the Ethics Committee of the Medical Association of Hamburg, Germany (WF-018/20); written informed consent was obtained from all subjects.

Between January 2012 and July 2019, all newly signed players from one professional soccer club (first and second national league) underwent MRI of both knee joints during their “medical”. The MRI studies were performed routinely for newly signed players as part of the medical suitability examination. From this pool of 78 players, 69 male professional soccer players were included in the study. The inclusion criteria were that the player was at least 18 years old at the time of examination and that a complete 3.0-T MRI of both knee joints with high quality and resolution was available. The main exclusion criterion was the lack of a complete MRI of both knees, mainly due to time issues (*n* = 9). Further exclusion criteria were knee injury or knee surgery within the last 6 months. During the mentioned period of more than 7 years, no player had to be excluded due to (reported) severe knee problems at the time point of their “medical” (*n* = 0). Before the examination (which took place during the “medical” and on the same day as the MRI), all baseline data (age, height, weight and playing position) were obtained. Reported knee injuries and related days missed due to injury were documented based on the medical history and on available data from “Transfermarkt” (http://www.transfermarkt.com). When a player who is under contract in one of the larger European leagues misses one or more matches due to an injury, this injury is reported by “Transfermarkt” based on different sources [14, 25]. These media-reported knee injuries were matched with those recorded by player anamnesis and medical history. Additionally, the players were grouped according to the following positions: (1) goalkeeper; (2) defender; (3) midfielder; and (4) offensive player and striker.

The mean age of all 69 players was 23.8 ± 3.9 years. There were five goalkeepers, 19 defensive players, 20 midfielders and 25 offensive players that could be included in the study. Based on the available documentation (anamnesis, medical history, “Transfermarkt”), 22 of the 69 included players suffered a relevant knee injury in the past (including ligament, tendon, meniscus and cartilage injuries with ≥ one missed game due to the knee pathology). Overall, a mean of 63.6 ± 160 (range 0–1105 days) days missed due to knee injuries were observed. For players with documented prior injuries, the meantime missed due to injury was 200 ± 232 (range 5–1105 days). The demographics of the studied soccer players are provided in Table 1. Athletes were split into two main groups (prior injuries and no prior injuries). To address a potential age bias when comparing players with and without previous injuries, a third age-matched subgroup was randomly selected from the group of players without reported injuries.

**Table 1:** Demographics of studied soccer players

	Total (*n* = 69)	No prior injuries (*n* = 47)	Prior injuries (*n* = 22)	No prior injuries, age-matched (*n* = 26)
Age	23.8 (± 3.9)	22.6 (± 3.4)	26.4 (± 3.7)	24.6 (± 2.7)
Playing position	5^a^ 19^b^ 20^c^ 25^d^	4^a^ 12^b^ 15^c^ 16^d^	1^a^ 7^b^ 5^c^ 9^d^	4^a^ 8^a^ 7^a^ 7^a^
Days missed due to injury	63.6 (± 159.3)	0	199.5 (± 232.0)	0
WORMS	13.9 (± 13.3)	10.1 (± 8.3)	22.1 (± 17.7)	8.9 (± 4.4)

Values for age, days missed due to injury and the WORMS (mean of both knees) are expressed as the mean with SD. The playing position data are displayed as absolute numbers. Athletes were further split into two main groups (prior injuries and no prior injuries). To address a potential age bias when comparing players with and without previous injuries, a third age-matched subgroup was randomly selected from the group of players without reported injuries

^a^Goalkeeper

^b^Defense

^c^Midfield

^d^Offense

### Imaging

All MRI scans were acquired using a 3.0-T MRI system (Ingenia, Philips, Best, The Netherlands) with a dedicated 16-channel knee coil (dStream, Philips, Best, The Netherlands) at maximum knee flexion of 5°–10° Widely established sequences were performed [coronal T1-weighted fast spin-echo (T1-FSE) and transverse, sagittal, and coronal fat-suppressed proton-density fast spin-echo (fs-PD-FSE)] (Table 2).

**Table 2:** MRI sequences at 3.0 T with a 16-channel knee coil

Imaging parameters	T1-FSE coronal	fs-PD-FSE sagittal	fs-PD-FSE transverse	fs-PD-FSE coronal
TR (ms)	929	6549	7833	5449
TE (ms)	11	27	27	27
Acquisition matrix	600 × 345	408 × 281	520 × 310	400 × 294
Plane spatial resolution (mm^2^)	0.2 × 0.2	0.25 × 0.25	0.25 × 0.25	0.25 × 0.25
FOV (mm)	120	142	130	120
Slice thickness (mm)	2.0	2.5	2.0	2.5
Imaging time (min:s)	4:50	12:27	5:29	5:59
Total imaging time (min:s)	28:45			

*FSE* fast spin-echo, *fs* fat-suppressed, *PD* proton density, *TR* repetition time, *TE* echo time, *FOV* field of view

### Image analysis

One hundred thirty-eight knee MRI scans (both knees of 69 players) were included in the study. All images were reviewed on a dedicated PACS workstation (PACS IW, GE Healthcare). Image analysis was performed by one expert with 19 years of experience in musculoskeletal imaging and two radiologists with 5 years and 6 years of experience in musculoskeletal imaging. All raters were blinded to the name and possible previous injuries of the players. For reliability analysis, 20 cases were independently assessed by all three raters. The remaining studies were assessed by the two aforementioned radiologists.

### Quantification of MRI findings

The MRI findings of the knee joint were evaluated according to a slightly modified WORMS [17]. Since the original WORMS was developed for osteoarthritis (OA) of the knee joint, and we expected only mild degenerative changes, flattening or depression of the articular surfaces (bone attrition) was not evaluated. The scoring of cartilage morphological features and signal intensity, bone marrow oedema pattern, marginal osteophytes, synovitis/effusion, periarticular cysts/bursitis, anterior and posterior cruciate ligament integrity and medial and lateral collateral ligament integrity was performed as previously described [17]. The original divisions of the patella (medial and lateral), femoral condyles and tibial plateau (anterior, central and posterior) were analysed to determine the extent of regional involvement of cartilage changes, bone marrow oedema, subchondral cysts and osteophytes. For detailed information on the WORMS, see Supplemental Tables 1–3.

Other pathological conditions that were not included in the WORMS (e.g., bipartite patella, tendon abnormalities, fractures) were also noted.

Cartilage pathologies were graded with a score ranging from 0 (normal thickness and signal) to 6 (diffuse (> 75% of the region) full-thickness loss). Bone marrow oedema and subchondral cysts were graded from 0 (none) to 3 (present in > 50% of the region), whereas osteophytes were graded based on the WORMS with a score ranging from 0 (none) to 7 (very large). Alterations in meniscal signal intensity were assessed on a four-level scale based on the PD-weighted FSE sequence (0, normal; 1, minor radial or parrot beak tear; 2, nondisplaced tear or prior surgical repair; 3, displaced tear or partial resection; 4, complete destruction or complete resection). Both knees were evaluated independently, and the respective results were compared. The study was based on the mean WORMS for the left and right sides since no side-based differences were observed and available clinical data did not specify the exact injured knee. Furthermore, we chose this approach because a player’s performance is dependent on both knees.

### Statistical analysis

Statistical analysis was performed using SPSS software version 23 (SPSS Institute, Chicago, IL, USA) and Prism (GraphPad, San Diego, CL, USA) for Mac (Apple, Cupertino, CA, USA). Based on the sample of 138 knee joints (69 players), descriptive statistics were determined, including the mean and standard deviation (SD) and the median and range of the WORMS and days missed due to injury. Values > Q3 + 1.5 × interquartile range (IQR) were defined as outliers. Regarding test–retest reliability, intraclass correlation coefficients (ICCs) were determined based on the independent evaluation of 20 cases by three readers and all cases (*n* = 69) by two readers. The ICC estimates and their 95% confidence intervals (CIs) were calculated based on the mean rating (*k* = 2 and *k* = 3) and absolute agreement in a two-way mixed-effects model. Case–control matching was performed for age in SPSS with a match tolerance (fuzz factor) of “0”. Bivariate correlation analysis using the Pearson coefficient was performed for the WORMS and clinical data (age, playing position, previous injury and days missed due to injury). Multiple linear regression analysis was used to test whether the WORMS was a significant predictor of days missed due to injury. Furthermore, we tested whether demographic data significantly influenced the WORMS. Binary multiple logistic regression was performed to evaluate whether the WORMS could detect prior injuries.

To confirm the robustness of our key finding (“soccer players with prior injuries have a higher WORMS than uninjured players”), a post hoc power analysis was performed in SPSS (two-way *t* test, independent samples). The effect size was calculated based on our findings (Cohen’s *d*: 0.782). Assuming a significance of 0.05, our sample size delivered a statistical power of 0.87. *p* values less than 0.05 were considered statistically significant.

## Results

### Semiquantitative image analysis and correlations

There was no significant difference based on the respective side of the knee (left versus right) for all variables of the WORMS, with *p* values of 0.604–0.905 for cartilage, 0.129–0.870 for bone marrow lesions, 0.924–0.965 for cysts, 0.460–0.516 for osteophytes and 0.417–0.896 for menisci, ligament and synovial tissue. Hence, further data are provided for both knees together.

**Fig. 1:**
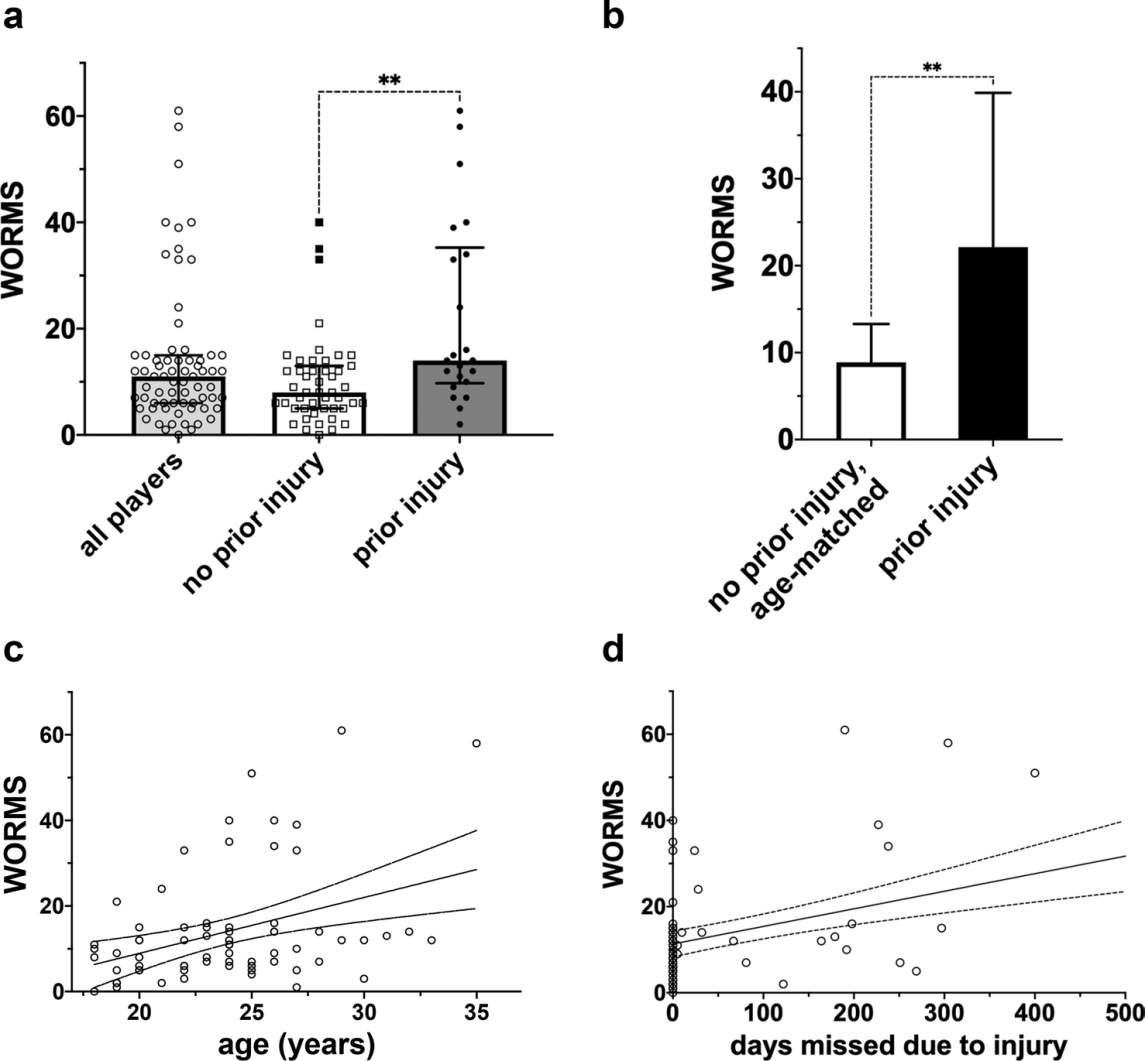
WORMS, correlation with age and days missed due to injury. **a** The median WORMS was 11 (9) in all players, 14 (26) in players with prior injuries and 8 (8) in players without prior injuries. Whiskers represent the IQR. Three outliers (> Q_3_ + 1.5xIQR) were identified in the group of noninjured players (indicated by solid squares, absolute scores: 33, 35 and 40). **b** The mean WORMS was significantly higher in previously injured players than in age-matched players without reported injuries (22.1 ± 17.7 vs. 8.9 ± 4.4, *p* < 0.002). Whiskers represent the SD. **c** The WORMS (all players) increased with age (*r*: 0.386, *p* < 0.001). Dashed lines represent the 95% CI. **d** The WORMS (all players) correlated with days missed due to injury (*r*: 0.489, *p* < 0.0001). Dashed lines represent the 95% CI. *Levels of significance

**Fig. 2:**
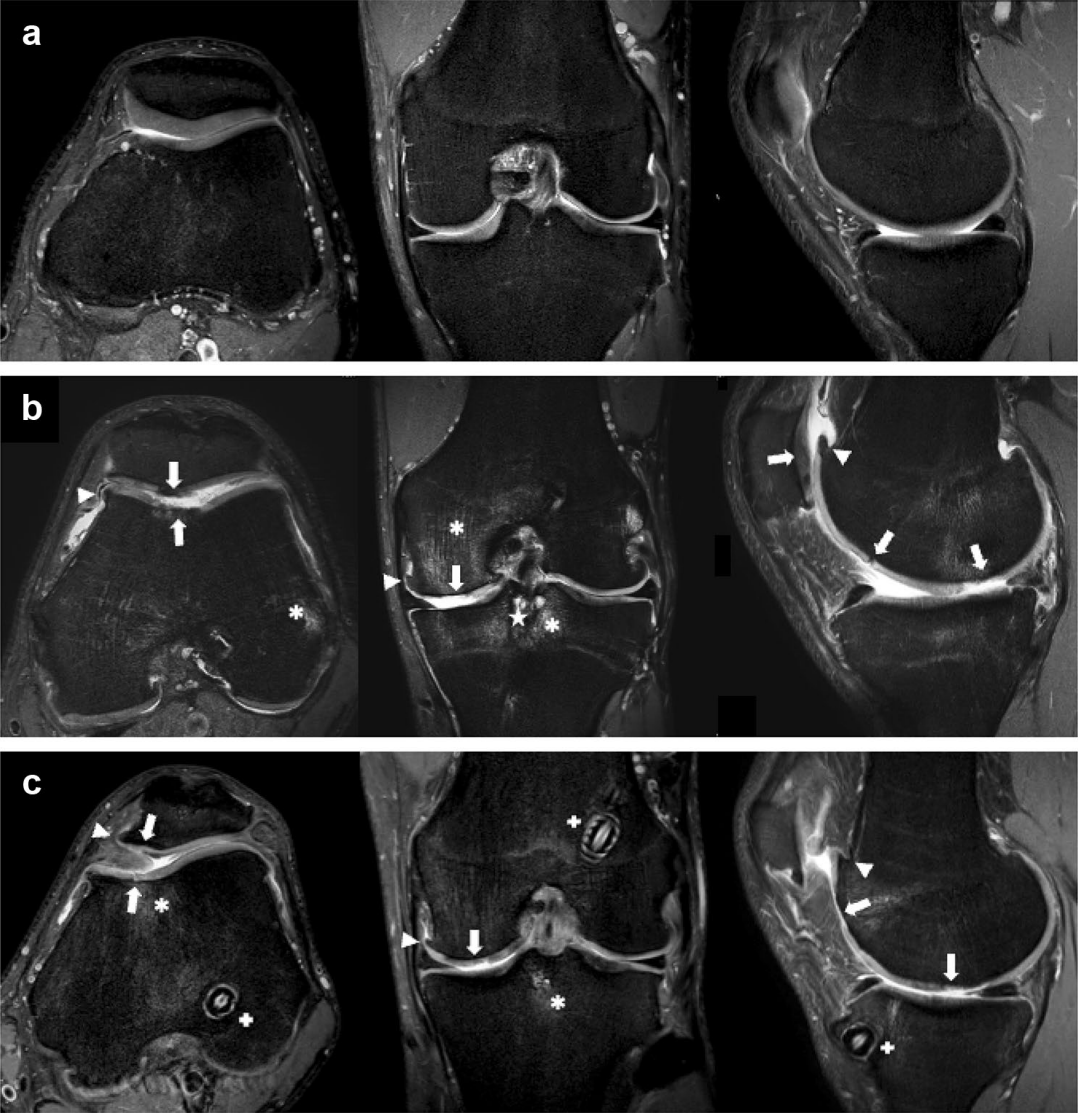
MRI of three soccer players (no prior injuries, prior injuries, no reported prior injuries). a Left knee of a 19-year-old striker with no prior injuries. The WORMS was 2 for both knees; hence, the total WORMS was 2. **b** Left knee of a 29-year-old midfielder with documented prior injuries. Note the severe cartilage damage, osteophytes and bone marrow oedema. The WORMS was 111 for the left knee and 10 for the right knee; hence, the total WORMS was 61. **c** Left knee of a 23-year-old defender with no documented prior injuries. The MRI demonstrates advanced joint damage and prior ACL repair. The WORMS was 53 on the left side and 27 on the right. The total WORMS was 40. Cartilage damage: arrows, bone marrow oedema: asterisk, osteophytes: arrowheads, cysts: star, ACL repair: plus sign

The median WORMS was 11 (9) in all players, 14 (26) in players with prior injuries and 8 (8) in players without prior injuries. Three outliers (> Q_3_ + 1.5xIQR) were identified in the group of noninjured players (absolute scores: 33, 35 and 40). The mean WORMS was 13.9 ± 13.3 (range 0–111) among all subjects, 10.1 ± 8.3 among players without reported injuries and 22.1 ± 17.7 among athletes with reported injuries. The mean WORMS was significantly higher among previously injured players than age-matched players without reported injuries (22.1 ± 17.7 vs. 8.9 ± 4.4, *p* < 0.002) (Fig. 1a, b). A comparison of three different knee joints is shown in Fig. 2.

The WORMS increased with age (*r*: 0.386, *p* < 0.001). Furthermore, the WORMS was significantly correlated with a prior knee injury (*r*: 0.424, *p* < 0.0001) and days missed due to injury (*r*: 0.489, *p* < 0.001) (Fig. 1c, d). No correlation was observed between the playing position and WORMS (*p* > 0.05).

### Interrater reliability

The ICC was 0.89 (0.87–0.91) for all three raters (*n* = 20) and 0.91 (0.90–0.92) for two readers (*n* = 69).

### Regression analysis

The first multiple linear regression model was created to test whether any of the available demographic and clinical data could predict the WORMS. The results demonstrated that prior injury and age explained 27% of the variance [*R*^2^ = 0.27, *F*(5, 66) = 9.11, *p* < 0.0001]. Prior injury was the only significant predictor of the WORMS (*β* = 11.11, *p* = 0.001). Age and playing position were not significant (n.s.) predictors.

**Fig. 3:**
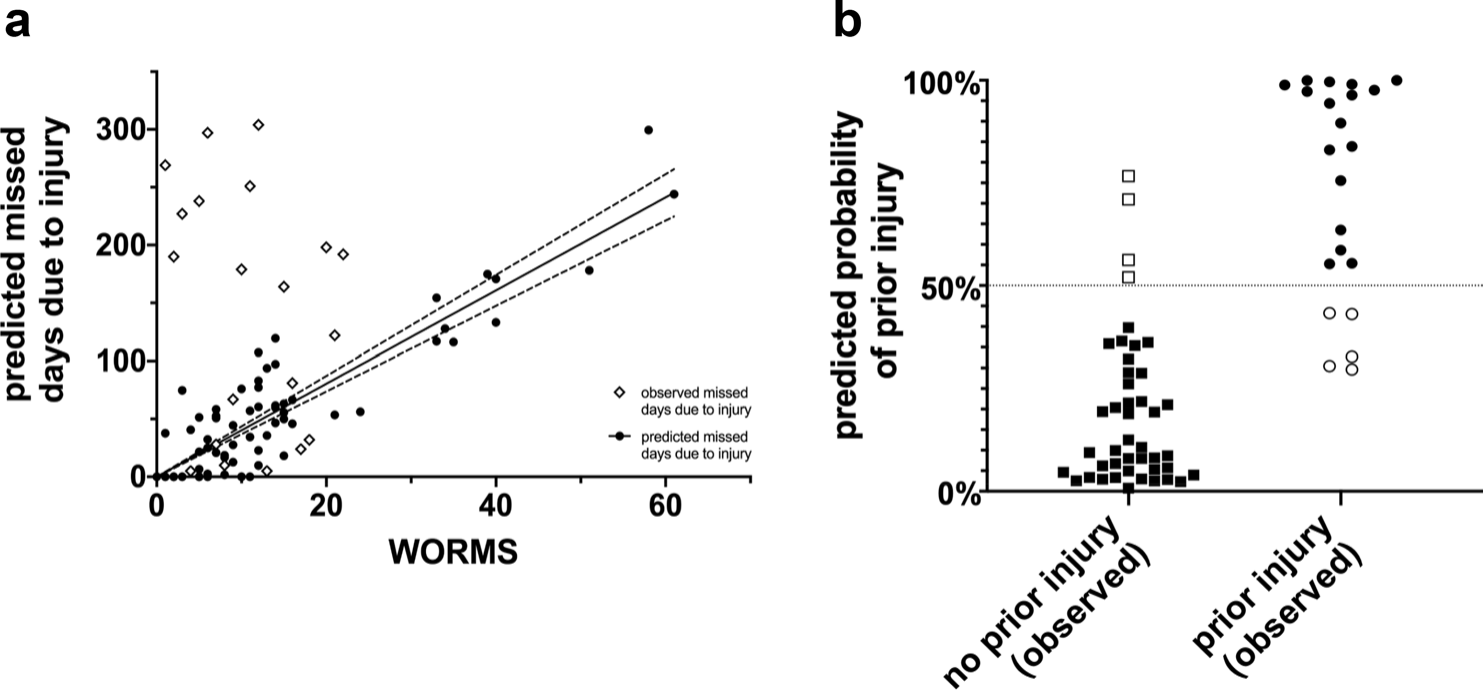
WORMS as a predictor for days missed due to injury and prior injury. **a** Multiple regression analysis was performed to test whether the WORMS, age and playing position significantly predicted days missed due to injury. The results of the regression indicated that the WORMS and age explained 38% of the variance [*R*^2^ = 0.38, *F*(5, 63) = 7.71, *p* < 0.0001]. The WORMS was a significant predictor of days missed due to injury (*β* = 3.40, *p* < 0.0002), as was age (*β* = 7.49, *p* = 0.013). The playing position was not a significant predictor (*p* = 0.794). Dashed lines represent the 95% CI. **b** Multiple logistic regression with prior injury as the outcome variable and the WORMS, age and playing position as predictors. The model had a positive predictive value for prior injury of 82%. The negative predictive value was 88% (sensitivity: 78%, specificity: 91%). The WORMS was a significant predictor of prior injury (*β* = 0.15 [0.06–0.28], OR = 0.86 [0.75–0.93], *p* = 0.006), as was age (*β* = 0.31 [0.11–0.54], OR = 0.73 [0.57–0.89], *p* = 0.004). The playing position had no significant influence (*p* > 0.35). Solid symbols indicate correctly predicted cases

The second multiple linear regression analysis was performed to test whether the WORMS, age or playing position significantly predicted days missed due to injury. The results of the regression indicated that the WORMS and age explained 38% of the variance [*R*^2^ = 0.38, *F*(5, 63) = 7.71, *p* < 0.0001]. The WORMS significantly predicted days missed due to injury (*β* = 3.40, *p* < 0.0002), as did age (*β* = 7.49, *p* = 0.013). The playing position was a n.s. predictor (Fig. 3c).

Binary multiple logistic regression was performed to investigate whether the WORMS could predict prior injuries (prior injuries vs. no prior injuries). The regression model had a positive predictive value for prior injuries of 82%. The negative predictive value was 88% (sensitivity: 78%, specificity: 91%). The WORMS was a significant predictor of prior injuries (*β* = 0.15 [0.06–0.28], OR = 0.86 [0.75–0.93], *p* = 0.006), as was age (*β* = 0.31 [0.11–0.54], OR = 0.73 [0.57–0.89], *p* = 0.004). The playing position had not a significant influence (n.s.) (Fig. 3d).

## Discussion

The key finding of our study was that soccer players with prior injuries showed higher WORMS than uninjured players. Furthermore, our findings demonstrate that semiquantitative knee MRI for WORMS determination during the soccer “medical” is a robust and reliable method for detecting prior trauma and that the WORMS correlates with previously missed days due to injury. The mean WORMS was significantly higher in previously injured players than in age-matched players without reported injuries. Three clear outliers were identified in players who had no documented prior knee injuries. The WORMS was correlated with age and prior injury. Regression analysis showed that the WORMS was predictive of prior days missed due to injury. Logistic regression showed that the WORMS has a high sensitivity (78%) and specificity (91%) for detecting prior knee injury. The playing position had no significant influence on joint health.

The fact that soccer players with prior injuries showed a significantly higher WORMS was confirmed by two separate methods: age matching and multiple linear regression. This observation laid the groundwork for all further analyses. Previously published studies by Roos et al., Driban et al. and Gouttebarge et al. strongly support our findings. These studies (based on conventional radiography or a questionnaire) found a higher prevalence of OA in professional soccer players with prior injuries than in players without prior injuries (33.3% vs. 10.7%) [4, 10, 21].

Furthermore, the WORMS was a significant predictor of previously missed days due to injury. A comparable study by Provencher et al. found that symptomatic focal chondral defects in National Football League (NFL) combine athletes were associated with poorer performance and loss of playing time [19]. Another related study showed that patients who suffered from injury-related OA and/or knee pain had a higher risk for reinjury on the ipsi- and contralateral sides [5]. This promising finding raises the need for future studies addressing whether the WORMS and/or its subcategories predict future loss of playing time.

Nevertheless, there is also a known high prevalence of focal chondral defects in asymptomatic athletes, and the natural history of chondral defects in the knee is not only based on prior injuries [9]. Furthermore, the degree to which these lesions will be symptomatic in the future and possibly reduce playing time is also unclear. In the present study, three players with no reported knee injuries or problems showed a highly increased WORMS of 33, 35 and 40 (as depicted in Fig. 1a). One of these players had undergone an unreported anterior cruciate ligament (ACL) surgery. The other two players had undergone no unreported surgeries; however, one player showed severe changes at the lateral meniscus, and the other player had severe chondral defects in the patellofemoral compartment. Although we found only three outliers, considering the possible consequences for the involved parties, routine MRI of the knee joints of all players during the “medical” can be recommended by the presented data.

As previously extensively demonstrated, the WORMS is a robust and reliable tool for semiquantitatively assessing the knee joint by means of MRI [2, 8, 12, 17, 20, 23, 24, 26]. In our study, it could be utilized for structured scoring and interpretation of the knee joints of professional soccer players.

In addition to the previous injury, the WORMS was correlated with age. This finding is concordant with those of previous studies that addressed longitudinal joint degradation due to the high physical demands of professional soccer [9, 18, 22].

As mentioned above, prior injuries were documented based on the anamnesis of each player during the medical examination and based on available medical reports and media-reported knee injuries (http://www.transfermarkt.com). There are existing studies showing that media-based injury data are valid, especially for severe injury types such as ACL injuries, with a validity of 100% [14]. Nevertheless, for all media-reported knee injuries together, the validity was only 78.2%, with a clear trend for reduced evidence of media analysis for less severe injuries [14, 25]. However, by pooling the media-reported data together with the player-reported data, the validity of a parameter such as “days missed due to knee-related injury” is as good as it can be.

There are other limitations to this study. First, exact information on previous injuries in former clubs is very often not available, other than the fact that a knee injury occurred. Hence, a more precise evaluation of the association of the WORMS with types of injuries and consecutive surgeries could not be performed. Furthermore, we did not evaluate the subcategories or subregions defined by the WORMS in our statistical analysis. This would go beyond the scope of this study and will be addressed in a follow-up evaluation. Finally, the number of included players was not very high, and in future studies, available data from different clubs could be combined. Nevertheless, this study has clear strengths. The subjects were all professional soccer players who underwent their initial medical examination at an elite soccer club. This process was highly standardized and well documented. Hence, baseline data for future studies are present for every single athlete. All players were examined with the same 3-T MRI scanner model, serving as a state-of-the-art tool for evaluating articular tissue.

## Conclusion

It was demonstrated by our results that semi-quantitative knee MRI is a reliable and robust method during the soccer “medical”. The WORMS detects prior trauma in players without documented injuries and correlates with previously missed days due to injury. Based on our findings, MRI during the “medical” is routinely recommended for players independent of any history of knee injury or knee problems.

## Supplementary Information

Below is the link to the electronic supplementary material.

Supplementary file1 (DOCX 27 KB)
