# Supplementary material for: Semi-quantitative magnetic resonance imaging scoring of the knee detects previous injuries in professional soccer players
Source: Knee Surg Sports Traumatol Arthrosc. 2022 Feb 19;30(4):1161–8. doi: 10.1007/s00167-022-06897-5 (PMC9007775; doi:10.1007/s00167-022-06897-5)
Supplement: Supplementary file 1 — Supplementary file1 (DOCX 27 KB) [file 167_2022_6897_MOESM1_ESM.docx]

| **Feature** | **Score** |
| --- | --- |
| **Cartilage** | 0 = normal thickness and signal  1 = normal thickness but increased signal on T2-weighted images  2.0 = parital-thickness focal defect <1cm  2.5 = full-thickness focal defect <1cm  3 = multiple areas of partial-thickness (grade 2.0) defects intermixed with areas of normal thickness, or a grade 2.0 defect wider than <75% of the region  4 = diffuse (≥75% of the region) partial thickness loss  5 = multiple areas of full-thickness loss (grade 2.5) or a grade 2.5 lesion wider than 1cm but <75% of the region  6 = diffuse (>75% of the region) full-thickness loss |
| **Bone marrow edema** | 0 = none  1 = <25% of the region  2 = 25-50% of the region  3 = >50% of the region |
| **Subchondral Cysts** | 0 = none  1 = <25% of the region  2 = 25-50% of the region  3 = >50% of the region |
| **Osteophytes** | 0 = none  1 = equivocal  2 = small  3 = small-moderate  4 = moderate  5 = moderate-large  6 = large  7 = very large |
| **Anterior and posterior cruciate ligament and medial and lateral collateral ligaments** | The ACL, PCL, MCL and LCL are independently scored as intact (0) or torn (1).  The sum of the ACL and PCL scores is added to half of the sum of the MCL and LCL scores. |
| **Lesions of the medial and lateral menisci** | Anterior horn, body, posterior horn are scored separately in medial, lateral meniscus from 0 to 4:  0 = no pathologies  1 = minor radial or parrot beak tear  2 = non-displaced tear or prior surgical repair  3 = displaced tear or partial resection  4 = complete maceration/destruction or complete resection |
| **Overall meniscus integretiy** | 0 = all 0  1 = at least one 1, but no >1  2 = 2 in only one region  3 = 2 in more than one region  4 = 3 in one or more region  5 = 4 in only one region  6 = 4 in more than one region |
| **Synovitis** | Synovial thickening and joint effusion are graded collectively from 0 to 3:  0 = normal  1 = <33% of maximum potential distention  2 = 33-66% of maximum potential distention  3 ≥66% of maximum potential distention |

**Table 1. Assessed features of the WORMS and the respective grading system.**

| **Anatomical region** | | **Plane used for scoring** | **Cartilage**  **(max.)** | **Bone marrow edema**  **(max.)** | **Subchondral cysts**  **(max.)** | **Osteophytes (max.)** |
| --- | --- | --- | --- | --- | --- | --- |
| Patella | medial | tra | 6 | 3 | 3 | 7 |
|  | lateral | tra | 6 | 3 | 3 | 7 |
| Medial femoral condyle | anterior | sag | 6 | 3 | 3 | 7 |
|  | central | sag | 6 | 3 | 3 | - |
|  | posterior | sag | 6 | 3 | 3 | 7 |
|  |  | cor | - | - | - | 7 |
| Medial tibial Plateau | anterior | sag | 6 | 3 | 3 | 7 |
|  | central | sag | 6 | 3 | 3 | - |
|  | posterior | sag | 6 | 3 | 3 | 7 |
|  |  | cor | - | - | - | 7 |
| Lateral femoral condyle | anterior | sag | 6 | 3 | 3 | 7 |
|  | central | sag | 6 | 3 | 3 | - |
|  | posterior | sag | 6 | 3 | 3 | 7 |
|  |  | cor | - | - | - | 7 |
| Lateral tibial plateau | anterior | sag | 6 | 3 | 3 | 7 |
|  | central | sag | 6 | 3 | 3 | - |
|  | posterior | sag | 6 | 3 | 3 | 7 |
|  |  | cor | - | - | - | 7 |
| Insertion of cruciate ligaments |  | cor | - | 3 | 3 | - |
|  | | | | | | |
| Medial meniscus |  | sag/cor | **tears, degeneration, prior surgical repair (max.)** | | | |
|  |  |  | 6 | | | |
| Lateral meniscus |  | sag/cor | 6 | | | |
| Tendons |  | sag/cor | 3 | | | |
| Synovitis |  | sag/cor/tra | **synovitits and cysts of the joint capsule** | | | |
|  |  |  | 3 | | | |
|  | | | | | | |
| **Whole knee (max.):** | **290** | | | | | |

**Supplemental Table 2.** **Detailed Display of the modified WORMS used in this study**.
With increasing degeneration or damage of the knee joint the score rises. A morphologically flawless knee joint received a score of 0. 290 points where the absolute maximum that could be achieved.

|  | **Joint** | **Anatomical subregions** |
| --- | --- | --- |
| **Whole knee** | patello-femoral joint (PFJ) | medial and lateral patella + trochlea |
|  | lateral femoro-tibial joint (LFTJ) | lateral tibial plateau + lateral femur condyle |
|  | medial femoro-tibial joint (MFTJ) | medial tibial plateau + medial femur condyle |

**Supplemental Table 3. Subjoints of the knee.**
